# Personalized Prevention of Cardiovascular Diseases Through Measurement of Plaque Burden in the Carotid Artery

**DOI:** 10.14740/cr2325

**Published:** 2026-10-22

**Authors:** Ansgar Adams, Michel Romanens

**Affiliations:** a BG Prevent GmbH Health Centre Koblenz, Koblenz, Germany; b Vascular Risk Foundation (Varifo), Olten, Switzerland

**Keywords:** Personalized medicine, Atherosclerosis, Cardiovascular risk, Carotid ultrasound

## Abstract

**Background:**

This study provides an overview of the results of carotid screening for the prevention of cardiovascular disease.

**Methods:**

Between 2009 and 2026, 11,540 individuals, 45% of whom were women, aged 20–65 years without clinical signs of cardiovascular disease, underwent carotid ultrasound examinations. Over 8,800 follow-up examinations were also performed. The total plaque area (TPA) and maximum plaque thickness were measured.

**Results:**

TPA was presented as percentile curves according to age and sex. In a second cohort, age-related TPA thresholds for coronary stenosis ≥ 50% were determined. Advanced atherosclerosis (type III/IVb) is associated with a high risk of cardiovascular disease. Early treatment with statins improves the prognosis of these patients. The individual course of the disease with rapid progression of plaque burden could not be predicted based on classic risk factors. Of 75 patients with advanced atherosclerosis who suffered a myocardial infarction or ischemic stroke, 35% had a low PROCAM risk (< 10%) and only 27% had a high PROCAM risk. Mortality (from any cause) was significantly lower in men treated with statins.

**Conclusions:**

Measuring plaque burden in the carotid artery allows for a good estimation of an individual’s risk of a cardiovascular event. In this non-randomized observational study, statin treatment significantly improved the prognosis in subjects with advanced carotid artery atherosclerosis (type III-IVb findings on ultrasound).

## Introduction

The prevention of cardiovascular disease (CVD) traditionally relies on the identification and treatment of risk factors. These include, in particular, elevated low-density lipoprotein (LDL) cholesterol, arterial hypertension, smoking, diabetes mellitus, and other factors. Established risk scores play an important role in population-based prevention. However, their main limitation is that they do not measure existing atherosclerotic disease, but rather estimate the future probability of events from risk factors. This can lead to discrepancies, especially in younger individuals [1]. A person may have a low calculated absolute risk due to their age and simultaneously exhibit an unusually high plaque burden for their age. The crucial question is therefore not only “What is the future risk?” but also “How much atherosclerosis is already present?” Carotid artery ultrasound offers a cost-effective, noninvasive, radiation-free, and relatively readily available method for this. By determining the total plaque area (TPA) and the maximum plaque thickness, the existing atherosclerosis can be objectively quantified. Regular follow-up examinations allow for monitoring the individual course of the disease and the efficacy of statin treatment. This study provides an overview of the results of ultrasound examinations in men and women without CVD from 2009 to 2026, as reported in several previous publications [2–7]. This approach is consistent with current recommendations on personalized prevention [1, 8, 9].

## Materials and Methods

Between 2009 and 2026, 11,540 individuals, 45% of whom were women, aged 20–65 years without clinical signs of CVD, underwent carotid ultrasound examinations. Over 8,800 follow-up examinations were performed, many of them involving repeated examinations of the same person. Plaque areas and maximum plaque thickness were determined in all individuals using the same methodology. The sum of all plaque areas was defined as the TPA. Based on this large population, age- and sex-specific percentile curves of the TPA were generated (Figs. 1, 2). This allows the plaque burden measured in a subject to be assessed not only in absolute terms, but also in relation to age and sex. In the 35- to 65-year-old age group, 12% of men and 4.2% of women showed advanced atherosclerosis (type III and IVb) (Fig. 3). All described investigations and evaluations on humans were conducted with the approval of the responsible ethics committee. The study was conducted in compliance with the ethical standards of the responsible institution on human subjects as well as with the Helsinki Declaration.

**Figure 1:**
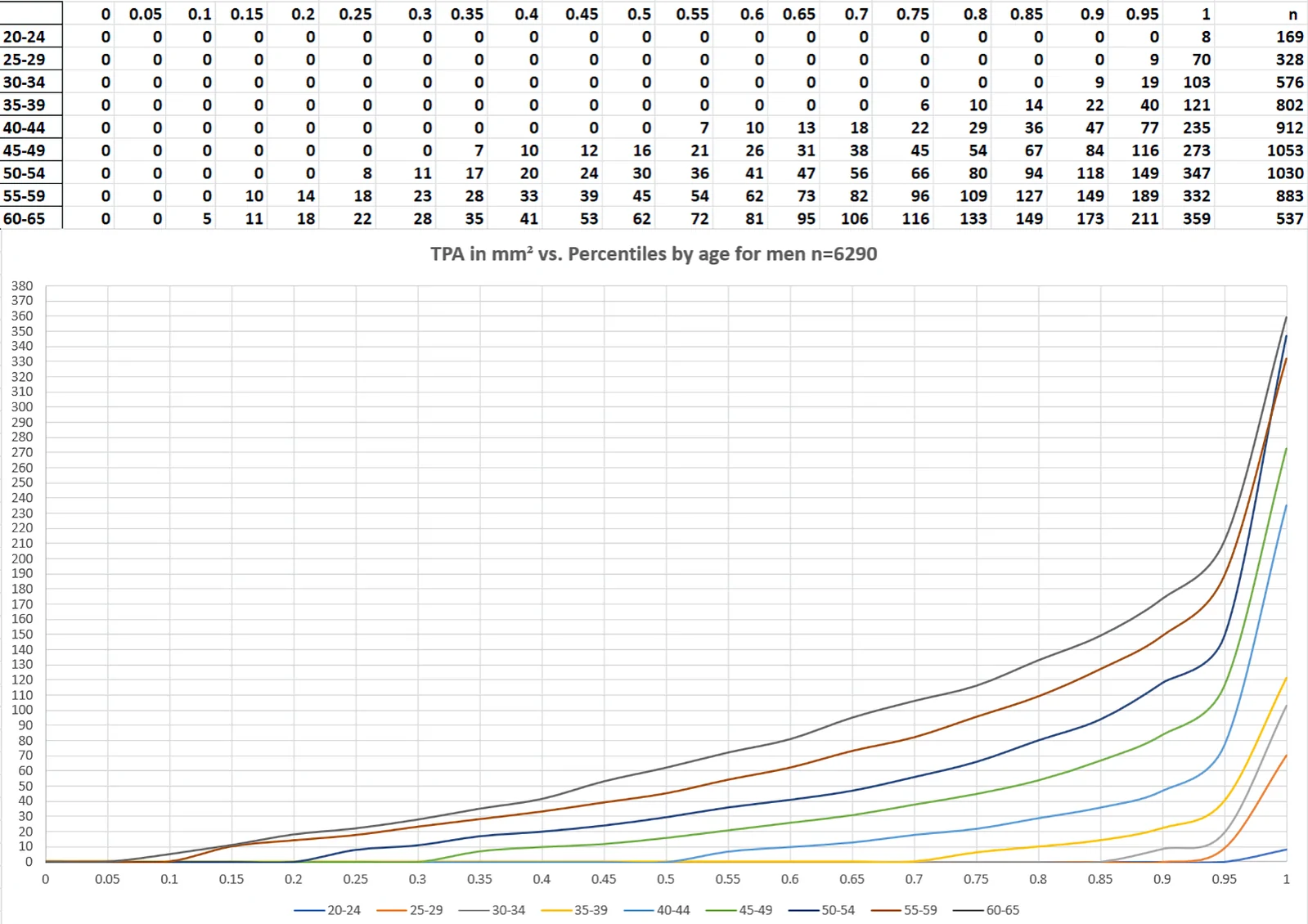
TPA percentiles by age for men. TPA: total plaque area.

**Figure 2:**
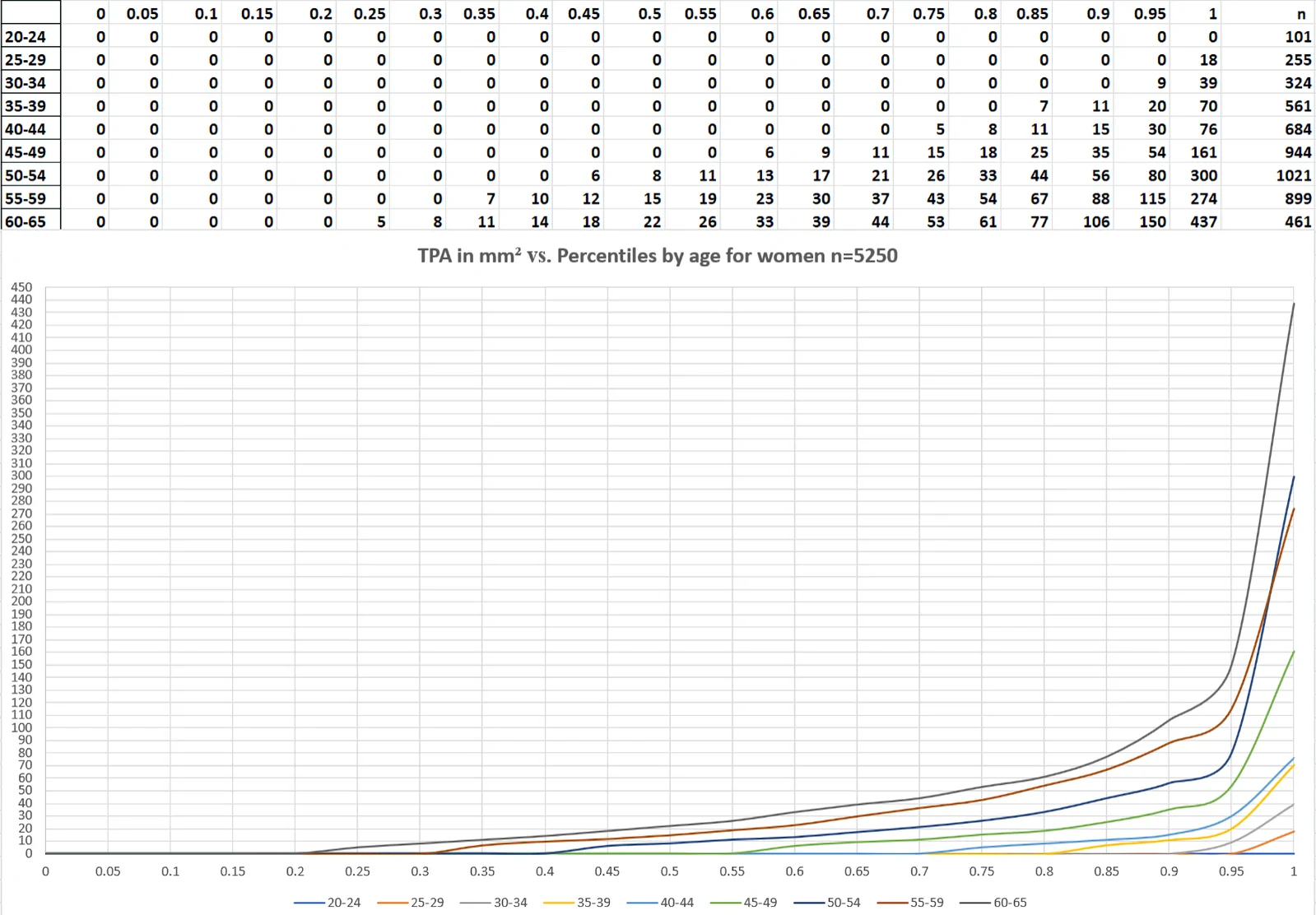
TPA percentiles by age for women. TPA: total plaque area.

**Figure 3:**
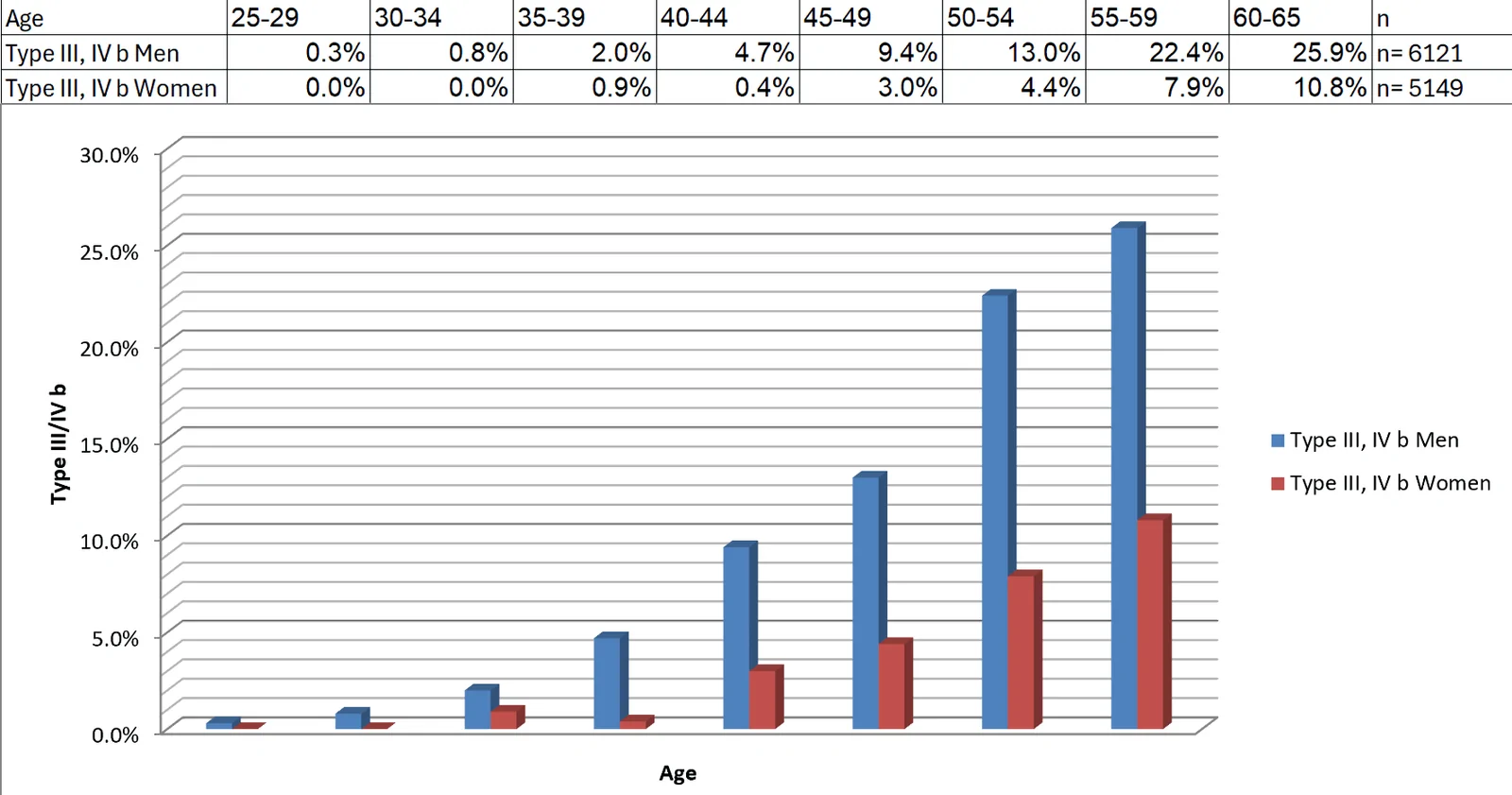
Proportion of men and women with advanced atherosclerosis.

### Independent validation

A classification of ultrasound findings was developed based on an independent patient group of 244 men and women aged 35–65 years, who underwent an ultrasound examination 1 day prior to scheduled coronary angiography. This classification was then adjusted for the age range of 35–65 years [2]. Low risk corresponds to ultrasound findings of types I and IIa, intermediate risk to those of types IIb and IVa, and high risk to those of types III and IVb. Type I is characterized by no or minimal atherosclerosis, with a TPA ≤ 24 mm^2^. Type IIa is characterized by flat plaques ≤ 2 mm, with a plaque area below the age-dependent threshold. Type IIb is characterized by flat plaques ≤ 2 mm, with a plaque area above the age-dependent threshold. Type III is characterized by a plaque with a maximum thickness ≥ 3.5 mm and a plaque area below the age-dependent threshold. Type IVa is characterized by mixed findings with flat plaques and plaques > 2 mm, but a plaque area below the age-dependent threshold. Type IVb is characterized by mixed findings with plaques > 2 mm and simultaneously a plaque area above the age-dependent threshold. The TPA thresholds used were as follows: age < 40 years, 60 mm^2^; 40–49 years, 80 mm^2^; 50–59 years, 110 mm^2^; 60–64 years, 130 mm^2^.

A maximum plaque thickness > 2 mm is considered abnormal; a thickness ≥ 3.5 mm defines pronounced plaque morphology, regardless of the TPA. The ultrasound examination was performed blinded prior to coronary angiography. The reference standard was defined as significant coronary artery disease (CAD) with a coronary stenosis > 50%.

The four-field table, adapted for age 35–65 years, yielded the following results: sensitivity, 81%; specificity, 76%; positive predictive value (PPV), 74%; negative predictive value (NPV), 83%; prevalence 45%.

In the asymptomatic population (4,340 healthy men and women age 35–65 years, with follow-up from 2009 to 2017), the sensitivity, adapted for the outcomes of major adverse cardiovascular events (MACE), coronary artery bypass grafting (CABG), percutaneous transluminal coronary angioplasty (PTCA), and coronary stenosis, was 80%, with a prevalence of 5% [7]. The comparable sensitivity despite the significantly different prevalence suggests a stable ability of the method to detect relevant disease.

According to the TPA cutoff values, the critical percentile for coronary stenosis in men was as follows: age 35–39 years: 96th percentile; age 40–44 years: 95th percentile; age 45–49 years: 89th percentile; age 50–54 years: 88th percentile; age 55–59 years: 80th percentile; age 60–65 years: 79th percentile. The critical percentile for coronary stenosis in women was as follows: age 35–39 years: 99th percentile; age 40–44 years: 99th percentile; age 45–49 years: 96th percentile; Age 50–54 years: 96th percentile; age 55–59 years: 94th percentile; age 60–65 years: 93rd percentile.

## Results

### Advanced carotid atherosclerosis as a marker of pre-existing CAD

The clinical significance of type III or type IVb findings was strongly supported by two further studies [3, 4]. In the study [4] “Early Diagnosis and Treatment of Coronary Heart Disease in Symptomatic Subjects With Advanced Vascular Atherosclerosis of the Carotid Artery (Type III and IVb Findings Using Ultrasound),” individuals with advanced carotid atherosclerosis who were asymptomatic at baseline and subsequently became symptomatic were examined. Further coronary diagnostics were performed using coronary computed tomography (CT) or coronary angiography. The second study [3], “Early Diagnosis and Treatment of Coronary Heart Disease in Asymptomatic Subjects With Advanced Vascular Atherosclerosis of the Carotid Artery (Type III and IVb Findings Using Ultrasound) and Risk Factors,” in contrast, examined individuals with advanced carotid atherosclerosis who remained asymptomatic at the time of examination. The coronary findings of both studies showed an almost identical distribution pattern.

Of the 106 individuals examined with advanced carotid atherosclerosis (type III/IVb), the following were present: smooth coronary arteries in 10.4%, coronary sclerosis in 13.2%, coronary stenosis of 30–50% in 22.6%, CAD with occlusion or three-vessel disease in 54.7%, and any coronary atherosclerosis in 89.6%. Thus, only 10.4% of the individuals had smooth coronary arteries.

Of particular significance is the finding that 54.7% of the patients had relevant CAD with stenoses > 50%, ranging from complete occlusion to three-vessel disease. Based on the therapeutic criteria used in the study, 89.6% of these patients—with the exception of those with smooth coronary arteries—would have been eligible for statin therapy. The vast majority of individuals with advanced carotid atherosclerosis already have coronary atherosclerotic disease. This supports the idea that the detection of advanced carotid atherosclerosis may represent more than just a risk modifier.

### Progression of atherosclerosis

In 198 men and 50 women (a total of 248 individuals) who initially presented with low to moderate plaque burden, advanced atherosclerosis developed over time [5]. The data have been updated. The mean follow-up time was 39 months (range, 5–96 months) for men and 37 months (range, 12–84 months) for women. Progression was not sufficiently predictable based on classic cardiovascular risk factors. The P values for the classic risk factors were > 0.05.

In men with documented progression, the mean TPA increased from 57 to 113 mm^2^, and the maximum plaque thickness increased from 2.1 to 3.0 mm. In women with documented progression, the mean TPA increased from 49 to 89 mm^2^, and the maximum plaque thickness increased from 2.2 to 3.3 mm. This demonstrates that atherosclerosis is a dynamic process whose rate can vary considerably between individuals. The longitudinal data show that a single examination does not fully capture the dynamics of the disease. The initial examination answers, “How much atherosclerosis is present?” A follow-up examination additionally answers, “How quickly is the disease progressing?” Since some individuals initially affected by low to moderate atherosclerosis developed advanced atherosclerosis within a few years, and this progression could not be adequately predicted by classic risk factors, a follow-up examination after approximately 3–5 years seems advisable. This would allow individuals with particularly rapid progression to be identified early and referred for a more intensive prevention strategy (Fig. 4).

**Figure 4:**
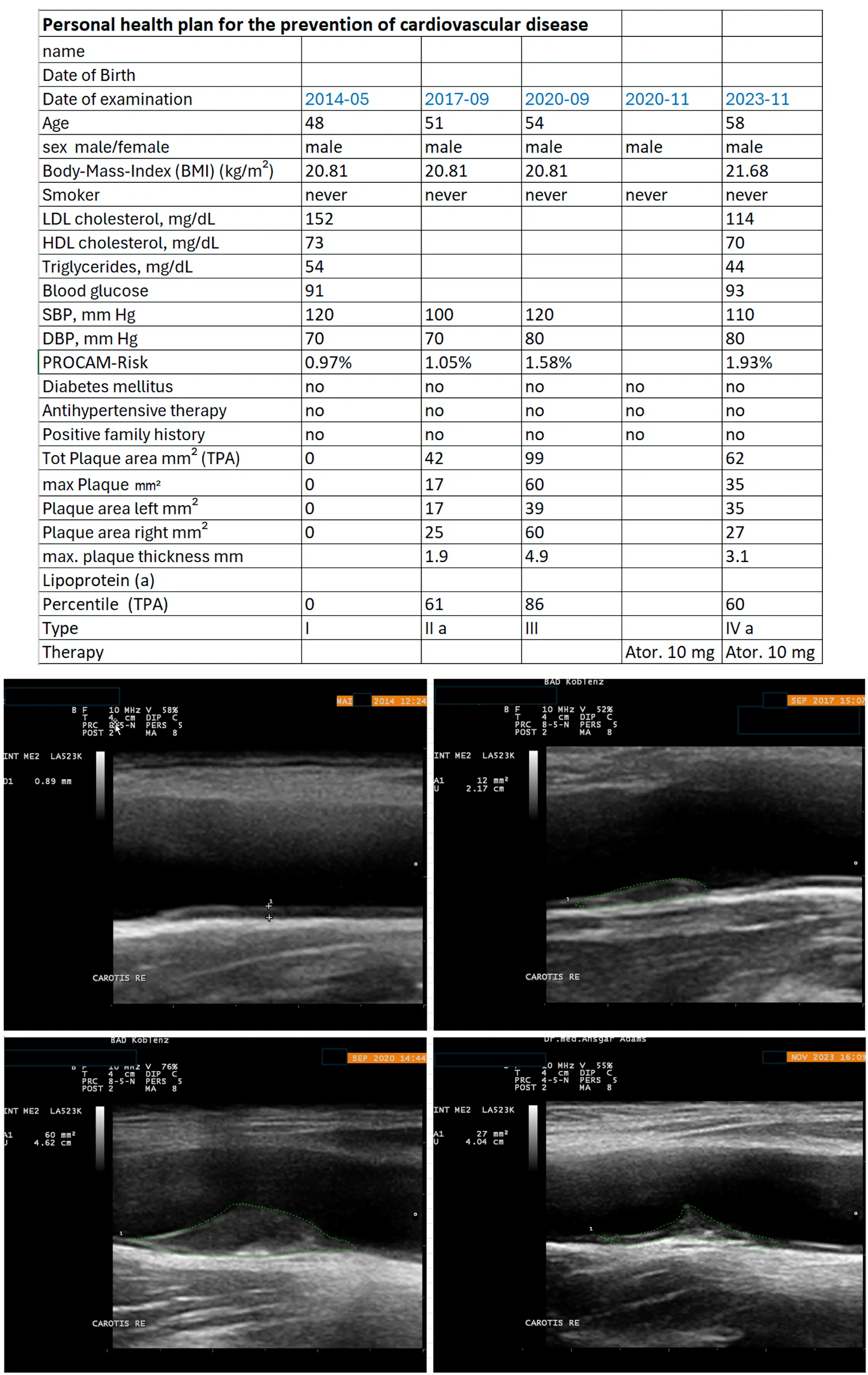
Example of the course of atherosclerosis. BMI: body mass index; DBP: diastolic blood pressure; HDL: high-density lipoprotein; LDL: low-density lipoprotein; SBP: blood pressure; TPA: total plaque area; Ator.: atorvastatin.

### Statin therapy and clinical outcome

The therapeutic importance of early treatment is further supported by studies [6, 7]. In individuals with advanced carotid atherosclerosis, statin therapy was associated with a significantly more favorable clinical outcome. The rate of MACE, PTCA, or CABG was 37.6% without statin therapy versus 16.1% with statin therapy. Mortality was also lower with statin therapy. However, these results are from an observational study. In the cited study [6], “Ultrasound examination of the carotid artery for improved prediction of cardiovascular events and the effect of statin treatment in advanced atherosclerosis: An observational study,” the predictive accuracy for cardiovascular events was compared between the PROCAM score and the measurement of plaque burden in the carotid artery. Plaque burden measurement was significantly superior to the risk score. Because of the lack of randomization, confounding and treatment selection cannot be completely ruled out. Therefore, the data provide strong evidence, but not definitive causal proof, of the therapeutic effect. Considering that a significant proportion of participants with advanced atherosclerosis already have CAD and that statins have been shown to reduce event rates, it is plausible that early drug therapy improves the outcome.

### Ethical aspects of a placebo-controlled study

The lack of randomization of the outcome data is a significant limitation. At the same time, the present results raise ethical considerations regarding a classic placebo-controlled long-term study. If 89.6% of individuals with advanced carotid atherosclerosis already have coronary atherosclerosis and 54.7% have relevant CAD, including three-vessel disease, this population cannot simply be equated with individuals without detectable atherosclerosis. A long-term placebo group would mean that individuals with objectively documented advanced atherosclerosis and a high probability of pre-existing CAD would not receive lipid-lowering therapy. This could be ethically problematic. This does not mean that randomized trials are unnecessary. Rather, future studies should use ethically sound randomized designs.

### An integrated concept of personalized prevention

The available data suggest a multi-stage approach: (1) determine risk factors: age, sex, LDL cholesterol, blood pressure, smoking, diabetes, and other factors determine the baseline risk; (2) directly detect atherosclerosis: carotid ultrasound shows whether atherosclerosis is already present; (3) quantify plaque burden: TPA and maximum plaque thickness determine the extent of the disease; (4) assess plaque burden according to age and sex: plaque burden is assessed using the corresponding percentiles; (5) identify advanced atherosclerosis: types III and IVb indicate a particularly pronounced plaque burden; (6) consider CAD: based on the available data, the possibility of pre-existing CAD should be considered in cases of advanced carotid atherosclerosis; (7) monitor progression: follow-up examinations show the individual rate of progression; and (8) individualize prevention: advanced or rapidly progressive atherosclerosis could be a reason to intensify prevention efforts.

## Discussion

The totality of the data suggests a shift in perspective on cardiovascular prevention. Classic risk scores estimate the probability of a future event. Imaging diagnostics, on the other hand, can detect existing anatomical disease. This distinction is particularly relevant in younger people. For example, a 40-year-old may have a low absolute 10-year risk due to their age, even though they have an unusually high plaque burden for that age. Conversely, a certain absolute plaque area may be less unusual in an older person. An absolute plaque area does not have the same significance for everyone. A TPA of 80 mm^2^ represents a significant deviation from the age-appropriate norm in a 40-year-old man, while the same plaque area may be considerably less unusual in a 60-year-old man (95th vs. 60th percentile). Age- and sex-specific TPA therefore allows for a more nuanced assessment than a single absolute threshold. TPA is therefore more appropriate for a 40-year-old man than a uniform absolute threshold. Even more significant is the observation that advanced carotid atherosclerosis is frequently associated with pre-existing CAD. The fact that 89.6% of the individuals examined had coronary atherosclerosis and 54.7% had relevant CAD, ranging from mild to severe, calls into question the classification of advanced carotid atherosclerosis as a mere “risk marker.” Rather, the data suggest that a type III or type IVb finding can be a marker of already advanced systemic atherosclerotic disease. The analysis of plaque burden by age and sex shows that in every age group from the age of 25 onwards, there are individuals with a significant plaque burden. This is not captured by the classic risk scores for young people. While the prevalence of coronary heart disease or severe CVD events is certainly low in the young population, the foundation for later events is laid as soon as the disease becomes visible.

In the cited study [2] “Prediction of coronary artery stenosis by measurement of total plaque area and thickness versus intima media thickness of the carotid artery,” it was shown that patients with clinical symptoms who underwent angiography due to suspected CAD all had smooth coronary vessels with a plaque burden of less than 14 mm^2^. It might be advisable to check whether the patient has a healthy, plaque-free carotid artery before performing coronary angiography. Figure 4 shows a reverse example. According to the risk score, there is a very low risk for a future CVD event. The imaging shows rapid progression of atherosclerosis, with a change to high risk and subsequent treatment success with only 10 mg of atorvastatin and good plaque regression.

### Conclusions

The totality of the available data supports an imaging-based approach for the early detection, quantification, and monitoring of atherosclerosis. The examination of 11,540 individuals allows for an age- and sex-specific assessment of carotid plaque burden. Independent, blinded validation in 244 individuals aged 35–65 years showed a sensitivity of 81%, a specificity of 76%, a PPV of 74%, and an NPV of 83%. In the asymptomatic population, the sensitivity was 80%. The two studies in individuals with advanced carotid atherosclerosis are particularly relevant. Here, 89.6% already showed coronary atherosclerosis, while 54.7% had significant CAD, ranging from arterial occlusion to three-vessel disease. Furthermore, the longitudinal data demonstrate that significant progression within a few years is possible and could not be adequately predicted by classic risk factors. A follow-up examination after approximately 3–5 years therefore seems advisable. Outcome data on statin therapy show a clear association between early treatment and a more favorable clinical course. However, due to the observational nature of the data, no definitive causal therapeutic effect can be inferred. At the same time, the high prevalence of CAD in individuals with advanced carotid atherosclerosis raises important ethical questions regarding long-term, placebo-controlled trials.

The central conclusion is therefore: The detection of advanced carotid atherosclerosis should perhaps not be considered solely as a risk modifier. Rather, it may indicate already advanced systemic atherosclerotic disease, which can be associated with a significant coronary disease burden even in clinically asymptomatic individuals. This results in a potential paradigm shift: from estimated risk to measured disease. Carotid ultrasound could offer a way to detect atherosclerosis, quantify it, assess it in relation to age and sex, monitor its progression, and personalize prevention accordingly.

### Limitations

This is a retrospective observational study and not a randomized placebo-controlled study, and this may introduce bias. Selective groups were studied, and the effects of other interventions were not analyzed.

Despite their consistency, the results must be interpreted with several limitations. The validation cohort comprised 244 individuals and had a higher prevalence of relevant CAD than a general asymptomatic population. PPV and NPV are therefore population dependent. The outcome data for statin therapy were derived from an observational study. A definitive causal statement is therefore not possible. Ultrasound measurement is also dependent on image quality, the device used, and the examiner’s experience. Reliable TPA determination requires a high-resolution ultrasound system and appropriately trained examiners. These limitations underscore the need for further standardization of the method and prospective studies.

## Data Availability

Any inquiries regarding supporting data availability of this study should be directed to the corresponding author.

## References

[1] Nielsen RV, Fuster V, Bundgaard H, Fuster JJ, Johri AM, Kofoed KF, Douglas PS (2024). Personalized intervention based on early detection of atherosclerosis: JACC State-of-the-Art review. J Am Coll Cardiol.

[2] Adams A, Bojara W (2015). [Prediction of coronary artery stenosis by measurement of total plaque area and thickness versus intima media thickness of the carotid artery]. Herz.

[3] Adams A, Bojara W, Schunk K (2018). Early diagnosis and treatment of coronary heart disease in asymptomatic subjects with advanced vascular atherosclerosis of the carotid artery (Type III and IV b Findings Using Ultrasound) and risk factors. Cardiol Res.

[4] Adams A, Bojara W, Schunk K (2017). Early diagnosis and treatment of coronary heart disease in symptomatic subjects with advanced vascular atherosclerosis of the carotid artery (Type III and IV b findings using ultrasound). Cardiol Res.

[5] Adams A, Bojara W, Romanens M (2025). Progression of atherosclerosis and hypothesis on the relationship between microvascular dysfunction, cardiovascular risk, and cancer. Cardiol Res.

[6] Adams A, Bojara W, Romanens M (2024). [Ultrasound examination of the carotid artery for improved prediction of cardiovascular events and the effect of statin treatment in advanced atherosclerosis: An observational study]. Herz.

[7] Adams A, Bojara W, Romanens M (2026). Prevention of cardiovascular disease and cancer through early statin treatment in advanced atherosclerosis: an observational study. Cardiol Res.

[8] Bundgaard H, Garcia-Lunar I, Kofoed KF, Hasselbalch R, Perez-Garcia CN, Kunkel JB, Staeger FF (2026). Prevalence of silent atherosclerosis across adult life. N Engl J Med.

[9] Chaiter Y, Fink DL, Machluf Y (2026). Ultrasound in cardiovascular care: a perspective on preventive, diagnostic, and monitoring applications. Front Cardiovasc Med.

